# Improving equity, efficiency and adherence to referral in Pakistan's eye health programmes: Pre- and post-pandemic onset

**DOI:** 10.3389/fpubh.2022.873192

**Published:** 2022-07-22

**Authors:** Asad Aslam Khan, Khalid Iqbal Talpur, Zahid Awan, Sergio Latorre Arteaga, Nigel M. Bolster, Marzieh Katibeh, Elanor Watts, Andrew Bastawrous

**Affiliations:** ^1^College of Ophthalmology and Allied Visual Sciences (COAVS), Mayo Hospital, King Edward Medical University, Lahore, Pakistan; ^2^National Committee for Eye Health, Government of Pakistan, Islamabad, Pakistan; ^3^Sindh Institute of Ophthalmology and Visual Sciences (SIOVS), Liaquat University, Jamshoro, Pakistan; ^4^Head of Inclusive Eye Health Projects, CBM Pakistan Country Office, CBM International, Islamabad, Pakistan; ^5^Peek Vision, London, United Kingdom; ^6^Department of Optometry, Faculty of Health Sciences, Lúrio University, Nampula, Mozambique; ^7^International Centre for Eye Health, Clinical Research Department, London School of Hygiene and Tropical Medicine, London, United Kingdom; ^8^Tennent Institute of Ophthalmology, Glasgow, United Kingdom

**Keywords:** digital health, mHealth, referral, attendance, eye health, visual impairment (VI), screening, vision

## Abstract

**Background:**

Over one billion people worldwide live with avoidable blindness or vision impairment. Eye Health Programmes tackle this by providing screening, primary eye care, refractive correction, and referral to hospital eye services. One point where patients can be lost in the treatment journey is adherence to hospital referral.

**Context:**

Peek Vision's software solutions have been used in Pakistan with the goal of increasing eye health programme coverage and effectiveness. This involved collaboration between health system stakeholders, international partners, local community leaders, social organizers and “Lady Health Workers”.

**Results:**

From the beginning of the programmes in November 2018, to the end of December 2021, 393,759 people have been screened, 26% of whom (*n* = 101,236) needed refractive services or secondary eye care, and so were referred onwards to the triage centers or hospital services. Except for a short period affected heavily by COVID-19 pandemic, the programmes reached an increasing number of people over time: screening coverage improved from 774 people per month to over 28,300 people per month. Gathering and discussing data regularly with stakeholders and implementers has enabled continuous improvement to service delivery. The quality of screening and adherence to hospital visits, gender balance differences and waiting time to hospital visits were also improved. Overall attendance to hospital appointments improved in 2020 compared to 2019 from 45% (95% CI: 42–48%) to 78% (95% CI: 76–80%) in women, and from 48% (95% CI: 45–52%) to 70% (95% CI: 68–73%) in men. These patients also accessed treatment more quickly: 30-day hospital referral adherence improved from 12% in 2019 to 66% in 2020. This approach helped to utilize refractive services more efficiently, reducing false positive referrals to triage from 10.6 to 5.9%. Hospital-based services were also utilized more efficiently, as primary eye care services and refractive services were mainly delivered at the primary healthcare level.

**Discussion:**

Despite various challenges, we demonstrate how data-driven decisions can lead to health programme systems changes, including patient counseling and appointment reminders, which can effectively improve adherence to referral, allowing programmes to better meet their community's needs.

## Introduction

### Eye health programmes

Over one billion people worldwide now live with avoidable blindness or visual impairment (VI), the majority in low- and middle-income countries (LMICs) or low-income socioeconomic groups (1). Eye health programmes aim to eliminate avoidable blindness, however their success depends upon effective implementation. Screening is frequently conducted in schools or the community. This step identifies whether the participants have any eye health problems warranting further care. The next steps include provision of primary eye care services and refractive correction, and referral to hospital eye services when needed. In community and school settings, most eye health needs consist of primary eye care and refractive services, which can be met by non-ophthalmologist personnel. The remaining problems which require referral are generally the most severe.

### The problem—adherence to referral

Previous studies have shown that community-based screening programmes, when they are delivered with digital solutions, can improve the utilization of eye care (2–4). However, in a model as described above, there are various stages where patients may be missed. They may not attend initial screening, may be lost to follow up at primary eye care/refractive services, may not attend hospital eye services following referral, or may not receive the recommended treatment. This leads to residual unmet need despite an otherwise high volume, effective screening programme. Therefore, when measuring the impact of eye health programmes, it is important to look beyond the number of people screened, as screening of visual impairment does not automatically translate into eye health outcomes (5).

For those with more complex eye care needs, one point in the patient journey where patients can be lost to follow up is adherence to hospital referral: patients failing to attend hospital ophthalmology appointments after a problem is identified. These patients are often those with the most serious ophthalmic problems, so failure at this stage can result in significant morbidity. Those who need hospital eye services require more resources to meet those needs, both from the patients' perspective (e.g., transport and time away from other obligations) (6) and health system perspective (e.g., professional cadre and surgical equipment). Therefore, there can be considerable barriers to attending a hospital appointment. Some previous studies have shown that the adherence to hospital referral after vision screening is low (around 30%) (4) even after provision of education, incentive packages, and subsidiary financial support (7). While these interventions do overcome some socioeconomic and logistic barriers, evidence-based approaches tailored to the local setting are needed to achieve an acceptable level of adherence and to ultimately improve vision (4, 7).

Commitment to hospital referrals is essential if eye health screening programmes are to be well accepted and integrated into existing local eye care systems, and have potential to provide effective outcomes. The inverse of this problem is inappropriate hospital attendance by patients who could have been successfully managed in the community, thereby inefficiently using hospital resources.

### Context

Pakistan was one of the earliest countries to adopt a National Eye Health plan, in 1993 (8, 9). However, the country has a substantial VI burden, with an estimated 26 million people living with vision loss in 2020, when near VI and mild distance VI are included (10). Prior to 2018, eye health services in Pakistan were mainly available at secondary care Tehsil Headquarter (THQ) Hospitals, such that the limited ophthalmologist resources were largely spent on simple problems which could be solved in the community.

Since 2018, an international non-governmental organization, CBM: Christian Blind Mission, has partnered with Peek Vision to use their proven public health methodologies supported by software (2, 11–13). These have been started in two areas in Pakistan: Talagang Tehsil in Chakwal district, Punjab province, and Matiari district, Sindh province.

In this paper, we will discuss an example of success in eye health programmes in Pakistan, overcoming the challenges through participatory processes and iterative improvements of the programme.

## Methods: Implementation of the programme

### Objectives

The objectives of the programme at initiation and during the iteration reviews were to:

- Increase coverage of eye health services
- Increase efficiency by reducing waiting times and workload of the health workforce
- Increase demand of services through community awareness
- Establish eye screening at Basic Health Units
- Integrate Optometrist services at Rural Health Centers connected to Ophthalmologist services at secondary Tehsil Headquarter (THQ) Hospitals
- Help managers to analyse performance, track patients through the services, identify those that are not reached and intervene to reduce barriers, and do so on a regular basis following a participatory process.

Key metrics in the programmes were:

- Number screened
- Number referred to triage
- Adherence to triage referral, i.e., attendance to primary eye care and refractive services
- Number referred to hospital
- Adherence to hospital referral, i.e., attendance to hospital eye services following referral.

**Figure 1:**
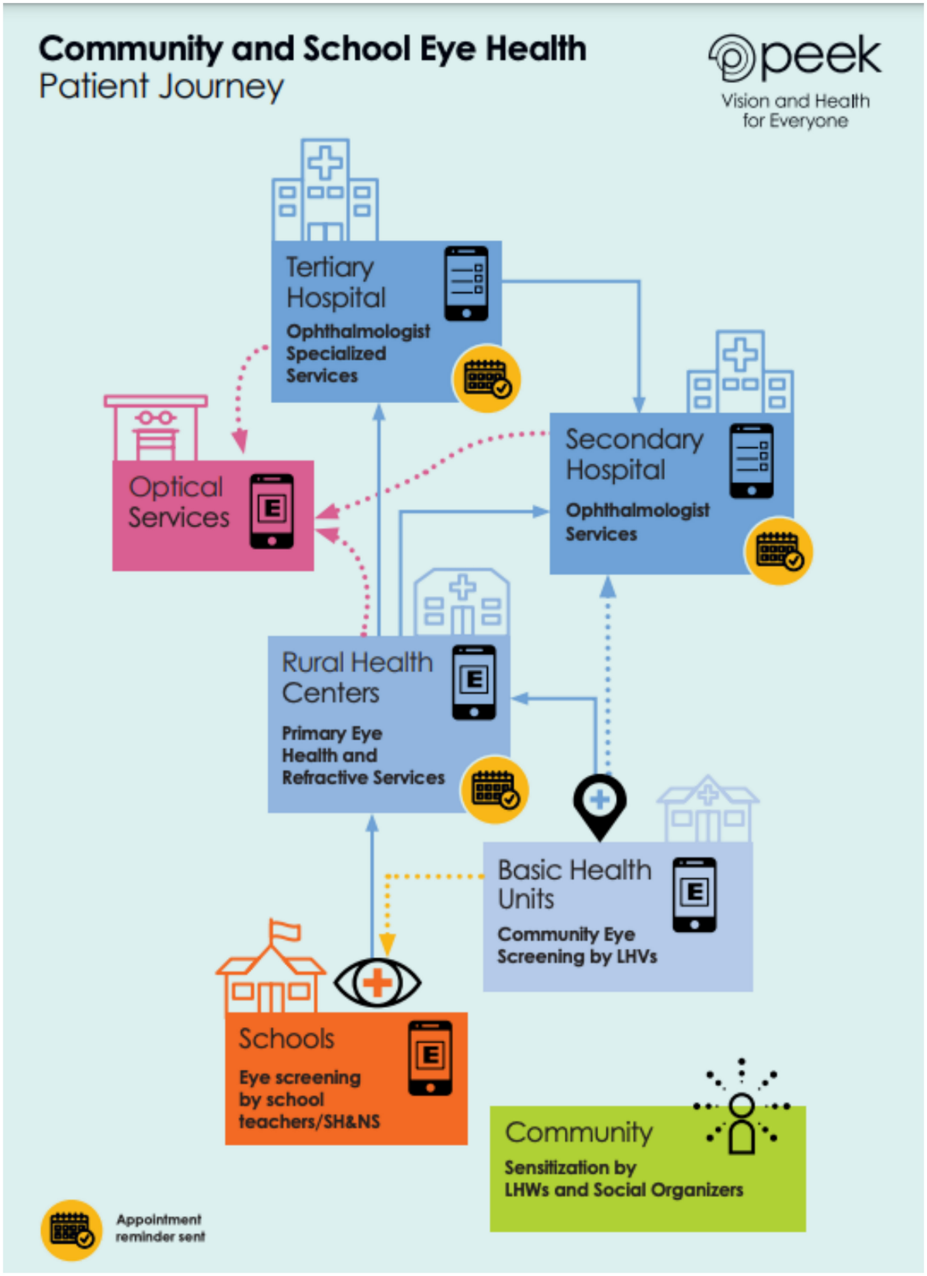
The patient journey approach and referral pathway in the Pakistan eye health programmes. LHWs, Lady Health Workers; LHVs, Lady Health Visitors; SH&NS, School Health and Nutrition Supervisors.

The community and school eye health programmes were tailored to local needs and resources, by local teams. Software tools, including smartphone-based vision tests, allowed data to be captured and transmitted digitally. The digital data collection and referral pathways were previously piloted in Kenya and later expanded in several other countries including Pakistan: the rationale and some methodological aspects have been explained in earlier publications (14). This enabled continuous monitoring of the effectiveness of eye health programmes, and a continuous improvement approach. These methods helped to identify hard-to-reach populations and to connect them to life-changing services. Adherence to hospital referral was identified as an area with potential for improvement. This was then targeted by social organizers, who increased community awareness of eye health and vision care. They also contacted patients who had been referred, to provide counseling regarding the importance of attending appointments, and to identify any obstacles to attendance. Allocation of appointments was also altered *via* a patient-initiated fixed appointments functionality, and appointment reminders sent *via* text message. The implementation involved collaboration between health system stakeholders in Pakistan, international partners, and local community health workers such as social organizers and Lady Health Workers (Figure 1).

### Timeline—rollout of the programmes

Initially, a situation analysis was carried out in April 2018. This included assessment of health facilities regarding human resources, infrastructure, accessibility, interfacility distances, and internet connectivity. Additionally, a population-based eye health survey using the Rapid Assessment of Avoidable Blindness (RAAB7) methodology, which utilizes Peek software, was conducted in Talagang from November to December 2018, alongside the launch of a Community Eye Health (CEH) programme in Talagang. The RAAB methodology has been described elsewhere (15, 16). The second iteration of this CEH programme was undertaken in September 2019, at the same time as the launch of the School Eye Health programme in the same region. In December 2019, a RAAB survey was undertaken in Matiari. The resulting data were used to design and plan a CEH programme which commenced in Matiari in September 2020. These programmes were led by the College of Ophthalmology and Allied Vision Sciences (COAVS) in Punjab province and the Sindh Institute of Ophthalmology and Visual Sciences (SIOVS) in Sindh province, in partnership with the Brien Holden Vision Institute and district authorities, and sponsored by CBM. ‘Lady Health Visitors', optometrists, dispensing opticians, and programme managers were trained to use Peek technology to register and track patients' progress from screening to diagnosis to treatment.

### Data sources, data security

RAAB7 and Peek School and Community software for data collection were used within these eye care programmes to monitor effectiveness and provide the data required to track adherence. Data storage, transmission and retrieval was in line with a Data Protection Agreement (DPA) with the local stakeholders, and followed the European Union General Data Protection Regulation (GDPR). Data was discussed regularly with stakeholders and implementers which enabled continuous improvement to service delivery.

## Results

### Screening coverage

In total, across the Punjab and Sindh Province programmes, the number of health facilities connected in this network increased from 3 in November 2018 (1 community screening Basic Health Unit, 1 triaging Rural Health Center, and 1 secondary eye health hospital) to 111 in 2021. This includes 84 Basic Health Units, 16 Rural Health Centers/City Hospitals, 8 THQ/District Hospitals and 3 specialized Tertiary Eye Hospitals. By the end of 2021, at least 108 Lady Health Visitors have been trained to screen using Peek.

School eye health programmes using Peek technology are being integrated into the CEH programmes in both provinces. The number of schools included within the screening programmes rose from 0 to 1567. This connected primary and secondary schools with their nearest health facilities, with the aim of reaching an estimated 500,000 children in Punjab and Sindh provinces.

**Figure 2:**
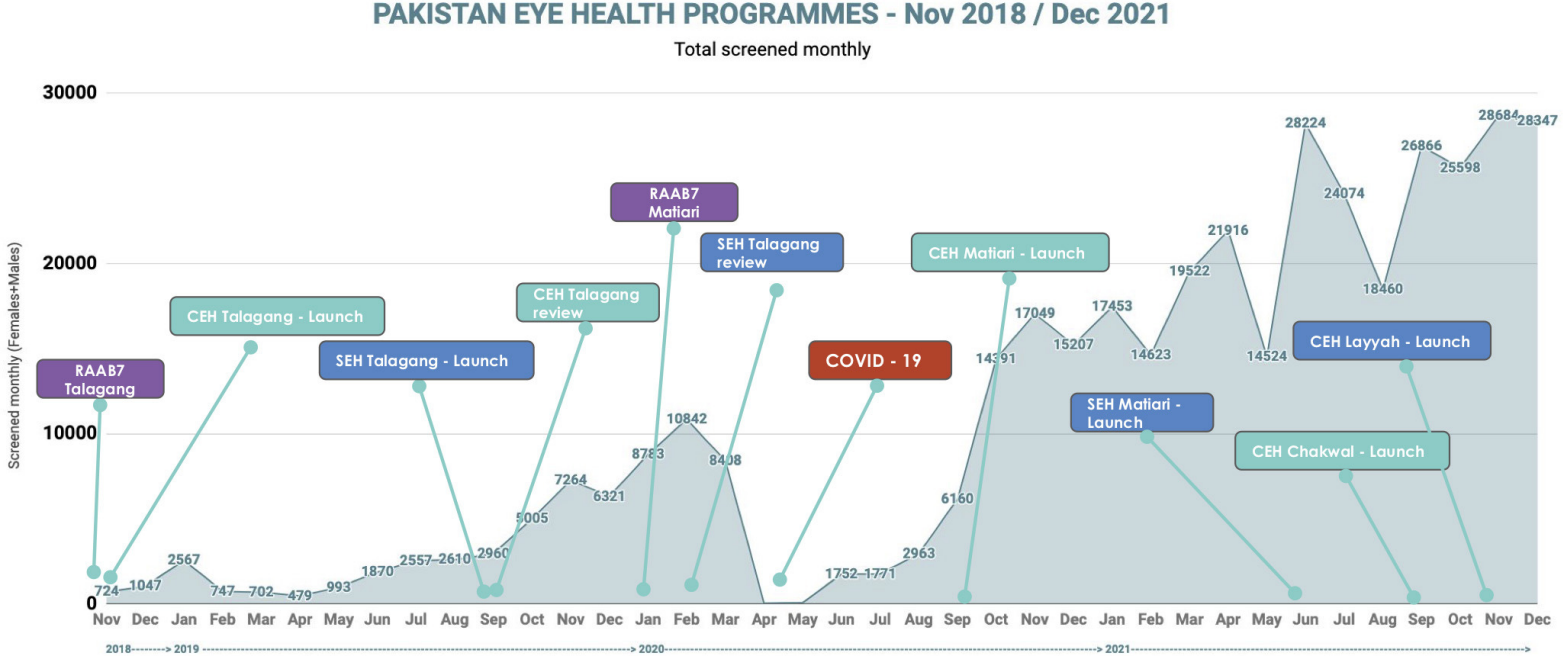
Screening volume, from programme initiation in November 2018 to December 2021. CEH, Community Eye Health; SEH, School Eye Health; RAAB7, Rapid Assessment of Avoidable Blindness, version 7 The chronological order of programme activities including RAAB surveys for evaluation of baseline prevalence of visual impairment, the launch of community and school eye health programmes, and iteration reviews for evaluation and monitoring of programmes are shown over time. Additionally, the number of people screened per month in all programmes is represented (blue line).

From the beginning of the programmes in November 2018, to the end of December 2021, 393,759 people had been screened, approximately 26% of whom (*n* = 101,236) needed refractive services or ophthalmology visits, and so were referred onwards to the triage centers or hospital services. Except for a short period affected heavily by the COVID-19 pandemic, the programmes reached an increasing number of people over time (Figure 2). Screening coverage improved from 774 people per month to over 28,300 per month. Women participated in screening more than men; however, gender balance in screening improved over time, resulting in the proportion of men accessing vision screening increasing from 18.1% in November 2018 to 33.1% in December 2021. These improvements were seen despite the pandemic.

### Programme outcomes: Met and unmet need

**Figure 3:**
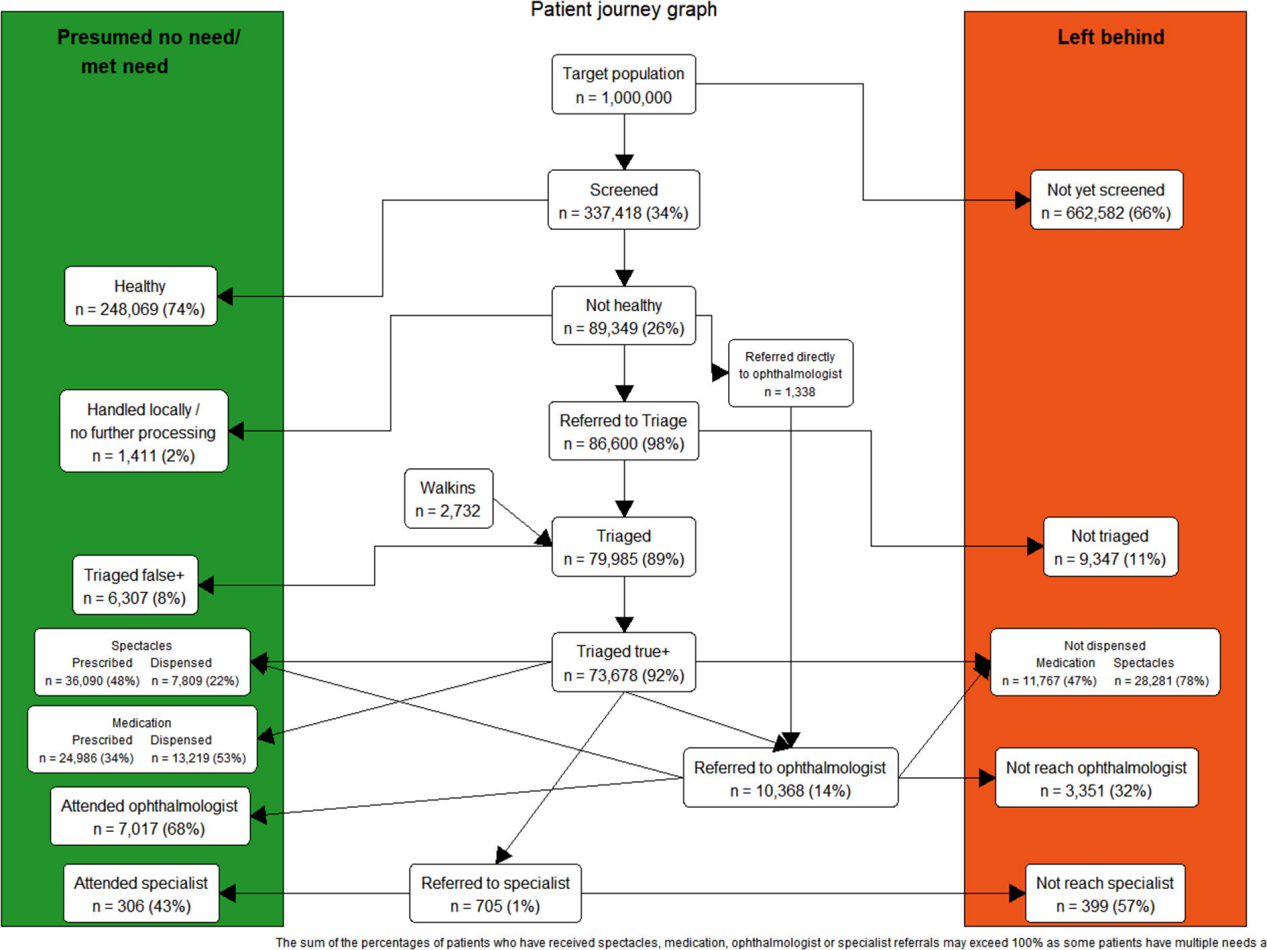
Patient Journey Graph: Total recruitment to different stages of community and school eye health programmes in Pakistan, November 2018 to September 2021. This figure shows the total recruitment and patient flow in different stages of the programmes. The programme aimed to reach 1,000,000 people, as defined during its design phase. During this time period, approximately one third of the target population were screened, 26% of whom were referred onwards for further assessment or treatment. 10,368 people were referred for review by an ophthalmologist. If someone has not yet reached hospital following referral, or not received spectacles or medication, they were included in the Left Behind column, showing that their needs are still waiting to be met. Where patients have received service (or have no need of treatment), they are included in the Presumed No Need/Met Need column. Of note, some patients currently have partially met need, e.g., those who have received a spectacle prescription but not yet had spectacles dispensed. In these cases, patients may appear in both columns. Information regarding medication was only gathered from triage centers where medications for primary eye conditions could be prescribed and dispensed.

Figure 3 shows the details of total recruitment of people in different stages of the programmes. As a longitudinal approach of patient outcomes is important in this figure, we have included data of those who were screened up to the end of September 2021. This is to allow at least a 3-month period for hospital attendance or spectacle dispensation. If someone has not yet reached the hospital, or received spectacles or medication, they were included in the unmet needs column showing that their needs are still waiting to be met. As illustrated in this figure, 337,418 people were screened from the start of the programme to the end of September 2021, 26% of whom were referred for further assessment or treatment. The percentage of true positives following referral to triage (those screened as needing further treatment who did indeed need further treatment) was 92%.

As shown in Figure 3, the majority of people who were referred to an ophthalmologist were referred after the triage stage, however, a small proportion (*n* = 1,338/10,368, 13%) were directly referred from the screening stage. If we follow recruitment flow from screening, only 0.3% of all those who were screened were referred directly to hospital and ophthalmology visits, and the others (2.6%) who needed ophthalmology visits were referred after triage. In total approximately 3% of the screened population had secondary eye care needs that required hospital visits.

Information about medication was only included from the triage centers where medications for primary eye conditions could be prescribed and dispensed. As shown, 34% of people who were triaged received medical prescriptions. Fifty three perecent of these prescriptions were dispensed on the project site. The remaining 47% may have either collected their medication elsewhere, or not yet had their medication dispensed.

The majority of spectacle prescriptions were provided at triage stage, while some were given following hospital visits. From data collected thus far, 36,090 persons (48%) were found to have refractive error and received a spectacle prescription. At least 7,809 persons (22%) have received their spectacles from the project sites to date.

### Adherence to hospital referral

**Figure 4:**
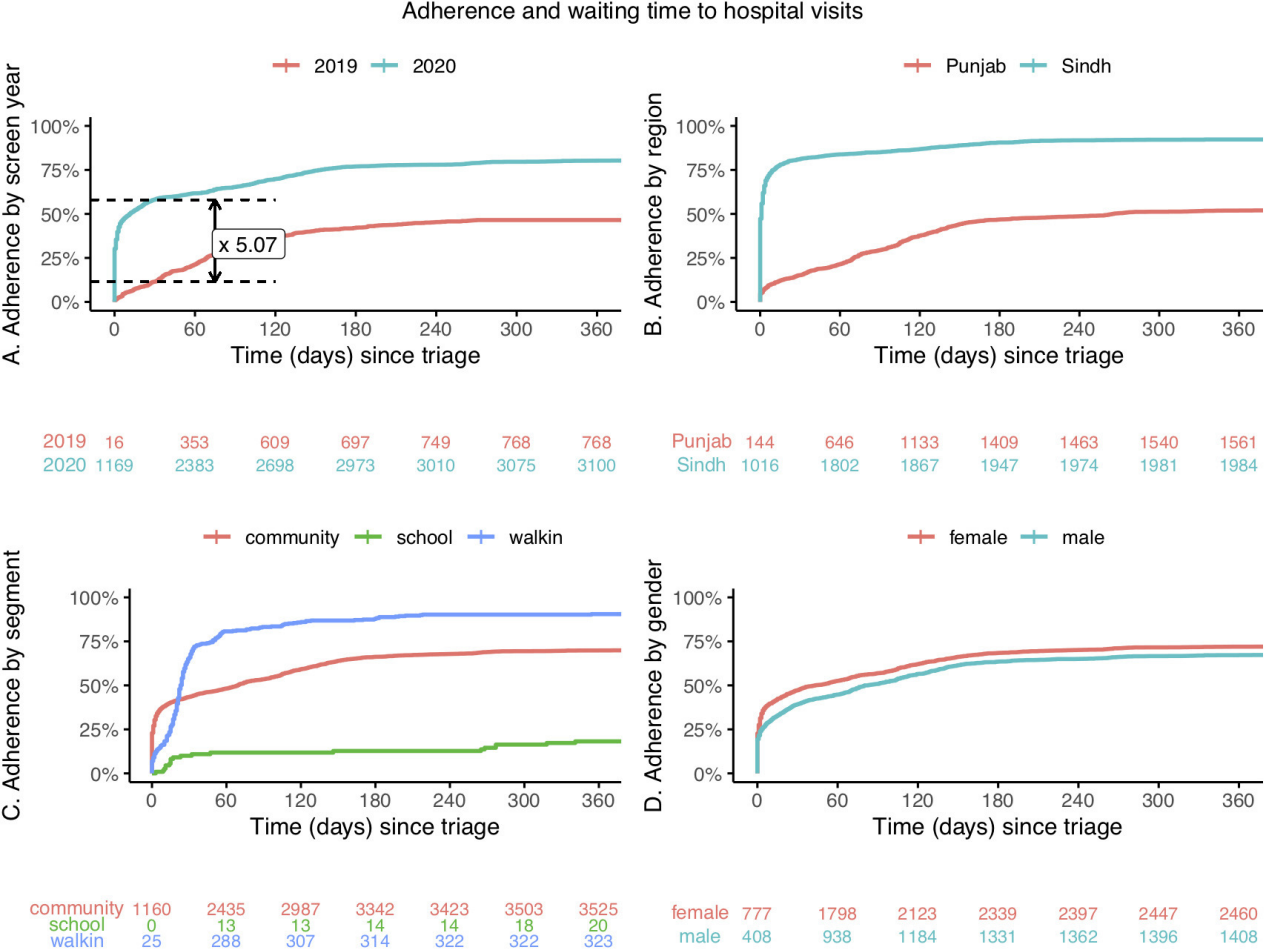
Sub-group analysis of cumulative adherence to hospital referral, and waiting time to hospital visit, in 2019 and 2020. **(A)** By screening year. **(B)** By region. **(C)** By source of referral: community screening, school screening, unscreened walk-in patients. **(D)** By gender Red, blue and green numbers below the graphs represent the cumulative number of attendees to hospital eye services following referral from triage.

Figure 4 shows sub-group analysis of adherence to hospital referral in 2019 and 2020, and waiting time to hospital visits. In addition it shows that 30-day hospital referral adherence improved by 5 times from 12% in 2019 to 66% in 2020 (Figure 4A), with a reduction in average waiting time to hospital appointments. Various other factors were shown also to affect adherence to referral. These included location of the programme, with larger improvements in Matiari in Sindh province (Figure 4B). Within each region, adherence varied with distance from the referral center, with lower attendance if not within walking distance of the hospital. In Talagang, early in the programme, >90% adherence to referral was seen amongst patients based within walking distance of the referral center. This is in comparison to 0–30% for those who required transport. There was also lower adherence to referral in younger school children (Figure 4C).

Attendance to hospital appointments was comparable between men and women (Figure 4D) and in both genders adherence improved in 2020 compared to 2019: from 45% (95%CI: 42–48%) to 78% (95%CI: 76–80%) in women, and from 48% (95%CI: 45–52%) to 70% (95%CI: 6–73%) in men.

### Quality of screening/appropriate referral

As mentioned above, the quantity (number screened) and quality of screening improved during the programme. True and false positives were used as a marking of screening quality, as this information could be collected during the programme. The proportion of false positive referrals from the screening stage to the triage stage was reduced from 10.6% in 2019 and 10.9% in 2020, to 5.9% in 2021. In particular, during the first half of 2021, an audit by location revealed some areas with an especially high rate of false positives, including one THQ hospital receiving 43% false positive referrals from screeners. The location analysis functionality revealed that most cases were referred from a specific facility, allowing the problem to be addressed with re-training. This, combined with increased communication between the Rural Health Center and the referring Basic Health Units, resulted in a reduction of false positive referrals from 86 to 17% at the Rural Health Center in question. In this subregion overall, false positive referrals improved from 43 to 4% by the end of September 2021.

In addition, hospital referrals for patients with refractive errors reduced from 41.2 to 1.2%, while the percentage of hospital patients attending with cataract increased from 6.7 to 50.1% in the first year of programme implementation at the THQ Hospital.

## Discussion

### Practical implications

Eye health programmes in Pakistan have expanded considerably over the last few years, with significant growth to the network of health facilities and schools. In this community case study, we have shown that modifications of the programme based on ongoing review of data and evidence can increasingly improve the programme, specifically attendance to hospital appointments which is an important step toward eye care utilization. In addition, over time, eye problems that were referred to the hospital included more complex, secondary care problems, and fewer primary eye care issues. The latter were instead identified and handled in the prehospital stages, reducing pressure on services, and unnecessary travel and potential anxiety for individuals (17). Since 2018 (when the programme started), fewer people are attending THQ hospital services with refractive error, and the proportion attending with cataract increased. Therefore, the implemented screening programme helped to utilize hospital-based services more efficiently, meaning that primary eye care services and refractive services were mainly delivered in the screening and triage levels, maintaining the capacity of hospitals for delivering secondary and tertiary eye care services.

Increasing the proportion of patients who attend hospital eye services following referral will have a range of downstream effects. The number of patients receiving treatment for their more complex disorders will increase, improving treatment coverage, such as Cataract Surgical Coverage. This would be expected to reduce the burden of VI in the region, or at least slow the current increase. The magnitude of this effect would be measurable in future RAAB surveys.

There will be effects not only for the patients, but for the healthcare providers. This significant shift in caseload profile could necessitate changes to the provision of hospital eye services, to ensure hospital ophthalmologists are able to fulfill the new demand.

### Strategies used

1. Digital data monitoring and visualization.

In this programme, a digital system was used to constantly monitor patient results and throughput, allowing identification of points at which patients were lost from the care pathway, and the effectiveness of attempts to improve patient retention, *via* iterative review.

2. Establishing and strengthening a referral pathway from community screening to primary and secondary/tertiary eye care.

Screening was undertaken in schools and the community, integrating screening services in Basic Health Units and schools. This was carried out by trained, non-specialist Lady Health Visitors and designated teachers in Sindh, and School Health and Nutrition Supervisors in Punjab. When necessary, patients were then referred directly to existing public health facilities, including optometrist services at Rural Health Centers and ophthalmologists at Tehsil Health Quarter hospitals with the goal of Integrated Person-Centered Eye Care, as recommended by the World Report on Vision (18).

3. Patient counseling and community education.

In this programme, screening was conducted in defined places and the contact information of participants was obtained and stored *via* secured and end-to-end encrypted software. Referral information could then be shared with community members or students' parents *via* text messages, and/or face-to-face interaction with local community health workers, such as social organizers and Lady Health Workers. For patients to attend hospital appointments, the perceived benefit of attending the appointment must outweigh the perceived costs and inconvenience of doing so. The likelihood of this can be increased by patient education regarding the purpose and importance of the appointment (19), and by support with any patient-specific difficulties in attending. Additionally, patient reminders can reduce the number of missed appointments resulting from patients simply forgetting (20), although the effectiveness of this has varied between programmes (21).

### Lessons learned

The ease of data management provided by Peek software allowed constant monitoring of the points at which patients were being lost from the treatment pathway. This in turn allowed introduction of relatively simple modifications which could successfully target these dropout points (hospital referral). The modifications as discussed included raising community awareness, patient counseling, and appointment booking and reminders. The significance of distance from hospital was demonstrated, and the addition of fixed appointment functionality led to increased adherence in more distant sites.

Another significant finding was that the gender imbalance seen in patients accessing the programme, with high adherence among women, was the inverse of that which is normally seen, given established gender inequities in access to eye care (18, 22–24). These differences are compounded by reduced access to eye care for women in LMICs, influenced in some regions by reduced control of family finances and freedom to travel alone. These issues lead to significantly lower cataract surgical coverage in women (25, 26). As such, in order to tackle Sustainable Development Goal (SDG) 5—Gender Equality—it is important to monitor how a programme is reaching each gender, and take necessary measures to make it easier for all patients to access assessment and treatment. In this programme, women not only consisted of a noticeably higher proportion of the people who were screened, but also their adherence to the hospital appointments was as high as men. The higher utilization of community based mHealth programmes has been shown previously (4), which shows when programmes are delivered close to the households, gender equity is achievable.

### Limitations

For optimal data collection, the RAAB surveys would have been completed prior to initiation of the Community Eye Health programmes. However, data were collected as part of a public health programme rather than a research study, and the intervention was not delayed for this purpose, so RAAB data was collected alongside the community programme.

Components of screening quality beyond true positive rates, including false negatives, could not be measured during these stages of screening and treatment. As more information is collected in future screening and follow up, a more in-depth assessment of screening will be possible.

There was a noticeable difference in adherence to referral between the two areas (Talagang/Chakwal and Matiari). It is important to acknowledge that in combining data from these two programmes, part of the improvement was due to higher adherence in the second programme from the offset. Various possible contributing factors have been suggested for this difference, including the second programme implementing lessons learnt from the first, e.g., optimal workflow with same day triage and referral, raising community awareness, and telephone appointment reminders. Shorter distance to referral site may also contribute to improved adherence in Matiari—most extremely demonstrated in centers where screeners, optometrists and ophthalmologists are all available in the same health facility.

We have reported our observation of the changes to healthcare provision during the pandemic. However, collection of data by the programme did not focus on analysis of the effects of COVID-19. The effect of the pandemic on healthcare seeking behavior and hospital attendance needs more investigation that was beyond the objectives of these programmes.

### Contextual factors and generalisability

Peek CEH programmes have been designed to meet community eye health needs. In this paper we have described a successful example of implementation, scale-up and continuation of eye health programmes in different regions of Pakistan. These findings, which are consistent in two different programmes, are promising and in favor of an optimistic approach to eye health for Vision beyond 2020.

Previously, it has been shown that using digital capacities enhances the utilization of existing professional eye care resources compared to traditional, paper-based methods: in Kenya, attendance to the ophthalmology services following a school screening programme was significantly higher in the smartphone-based screening group than in paper-based conventional screening (54% vs. 22%) (2). In Iran, mHealth methods (using digital tests and smartphone connectivity capacities) were shown to improve both coverage of screening and adherence to referral compared to conventional means, particularly among people from lower socio-economic groups (4). Part of this effect is due to provision and visualization of real-time eye health and delivery data that enhances targeted resource management and uptake *via* connectivity capacities, and part of it is due to flexibility and mobility of small and cost-effective technology packages that improves reach to more vulnerable groups.

The COVID-19 global pandemic has introduced challenges to health systems all over the world. Simultaneous increased burdens on healthcare services resulting from COVID-19 patients, pressures to reduce inter-personal contact of patients and staff to limit disease transmission, and redistribution of staff, have led to a decrease in patients accessing healthcare, including eye care (27). A systematic review estimated a reduction in healthcare utilization of approximately one third during the pandemic (28). These reductions are thankfully lesser among more severe health conditions. To manage the changes, there has been a surge in the use of remote tele-health and technology-based/mHealth interventions (29, 30). These have included remote appointments (telephone and video call consultations) and virtual appointments in which patients attend a hospital for some tests, and management decisions are made without face-to-face consultations between the patient and doctor. Although tele-healthcare has been most easily implemented in HICs (31), it is perhaps most needed in the LMICs, where 8 out of 10 people with vision loss live, many of whom cannot access the eye care they need (32). In these eye care programmes, while a reduction in attendance was seen in the beginning of the pandemic in 2020, improvements were still achieved overall, exemplifying the potential for successful use of mHealth in a LMIC.

## Conclusion

Our experience, particularly in the last three years in Pakistan programmes, shows that mHealth methods are relevant and have the potential to produce good results, even during a pandemic. The data provided highlighted programme-specific areas for improvement, such as adherence to hospital referral, and monitored the success of strategies used to tackle this. In addition, this work emphasized that continuity of eye health programmes is essential to produce better results over time, as programme teams can use ongoing evidence to constantly improve via scalable iterations.

## Data availability statement

The original contributions presented in the study are included in the article/supplementary material, further inquiries can be directed to the corresponding author.

## Ethics statement

Ethical review and approval was not required for the programme on human participants in accordance with the Local Legislation and Institutional Requirements. Written informed consent from the participants' legal guardian/next of kin was not required to participate in this programme in accordance with the national legislation and the institutional requirements.

## Author contributions

AB, AK, KT, ZA, SA, and NB contributed to conception, design, and implementation of the programmes. MK performed data analysis. EW and MK wrote the first draft of the manuscript. SA wrote sections of the manuscript. All authors contributed to manuscript revision, read, and approved the submitted version.

## Funding

These programmes were primarily funded by CBM International (Programme Funding) [project codes 3760-PK-MYP and 3760-SIOVS-MYP]. This work was also supported by the National Institute for Health Research (NIHR) (using the UK's Official Development Assistance (ODA) Funding) and Wellcome [grant reference 215633/Z/19/Z] under the NIHR-Wellcome Partnership for Global Health Research.

## Conflict of interest

The Peek Vision Foundation (09919543) is a registered charity in England and Wales (1165960) with a wholly owned trading subsidiary, Peek Vision Ltd. (09937174). AB is Chief Excecutive Officer (CEO) of The Peek Vision Foundation and Peek Vision Ltd. SA and NB are employees of Peek Vision Ltd. MK and EW are consultants for Peek Vision Ltd.

The remaining authors declare that the research was conducted in the absence of any commercial or financial relationships that could be construed as a potential conflict of interest.

## Publisher's note

All claims expressed in this article are solely those of the authors and do not necessarily represent those of their affiliated organizations, or those of the publisher, the editors and the reviewers. Any product that may be evaluated in this article, or claim that may be made by its manufacturer, is not guaranteed or endorsed by the publisher.

## Authors disclaimer

The views expressed are those of the authors and not necessarily those of Wellcome, the NIHR or the Department of Health and Social Care.
